# Severe Maternal Morbidity and near Miss-Events in Women with Heart Disease: Insights from a Cohort Study

**DOI:** 10.3390/diagnostics15121524

**Published:** 2025-06-16

**Authors:** Felipe Favorette Campanharo, Edward Araujo Júnior, Daniel Born, Gustavo Yano Callado, Eduardo Félix Martins Santana, Sue Yazaki Sun, Rosiane Mattar

**Affiliations:** 1 Department of Obstetrics, Paulista School of Medicine, Federal University of São Paulo (EPM-UNIFESP), São Paulo 04023-062, SP, Brazil; favorette@hotmail.com (F.F.C.); dudes.felix@gmail.com (E.F.M.S.); sueysun@gmail.com (S.Y.S.); rosiane.mattar@unifesp.br (R.M.); 2 Department of Cardiology, Paulista School of Medicine, Federal University of São Paulo (EPM-UNIFESP), São Paulo 04023-062, SP, Brazil; dborn@terra.com.br; 3 Albert Einstein Israelite College of Health Sciences, Albert Einstein Israelite Hospital, São Paulo 05653-120, SP, Brazil; gycallado@gmail.com

**Keywords:** high-risk pregnancy, cardiopathies, morbidity, near miss

## Abstract

**Background/Objectives:** The maternal mortality ratio is one of the global health indicators, and cardiopathies are the leading indirect causes of maternal deaths. Proper management of pregnant women with heart disease is crucial, as the severity of these conditions can lead to complications during the perinatal period. This study aimed to evaluate the rate of severe maternal morbidity and associated factors in pregnant women with heart disease. **Methods:** A retrospective cohort study was conducted at a referral hospital in São Paulo from 2008 to 2017, including pregnant women with heart disease who underwent procedures in the obstetric center (*n* = 345). Sociodemographic, obstetric, and pre-existing conditions were analyzed, along with life-threatening conditions, near-miss events, and maternal deaths. Heart diseases were classified according to the World Health Organization (WHO) guidelines, and health indicators were calculated using WHO-recommended formulas. The Chi-square test or Likelihood Ratio test (*p* < 0.05) was used to compare severe maternal morbidity among women with heart disease. **Results:** The mean age of participants was 29.1 ± 7.29 years; most were white (58.8%), had completed high school (37.9%), and were married (71.6%). The most frequent pre-existing conditions were hypertension (9.6%) and diabetes mellitus (9.3%). The mean gestational age at admission/delivery was 37 weeks. According to the WHO classification, most women were classified as “II/III” (31.6%). Life-threatening conditions included hemorrhagic complications (13.9%), hypertensive complications (5.8%), clinical complications (19.7%), and severe management conditions (31.6%). Near-miss events occurred in 6.4% of patients, with clinical criteria in 2.9%, laboratory criteria in 4.3%, and management criteria in 3.5%. The cesarean section rate was 51%. Patients classified as WHO III and IV presented more severe management conditions (*p* < 0.0001), and those in WHO IV had a higher occurrence of near-miss events (*p* = 0.0001). Maternal mortality was 0.9% (*n* = 3). **Conclusions:** The incidence of severe maternal morbidity was 25 cases (22 near-miss events + 3 maternal deaths), equivalent to 2.86 per 1000 live births, and was significantly associated with WHO classifications III and IV.

## 1. Introduction

The Global Strategy for Women’s, Children’s, and Adolescents’ Health (2016–2030) extends the goals of the Millennium Development Goals, aiming not only to eliminate preventable deaths but also to create an environment in which these groups can thrive in health and well-being [1].

In Brazil, as in many parts of the world, maternal mortality (MM) due to direct causes has declined in recent decades. However, the same trend has not been observed for deaths resulting from indirect causes, with cardiopathies representing the leading indirect cause of MM in high-income countries [2,3,4,5]. Although the prevalence of significant heart disease during pregnancy is relatively low (approximately 1–4%), it substantially increases the risk of adverse perinatal events—particularly maternal complications [6].

Pregnancy imposes dramatic changes on the cardiovascular system, including a 30–50% increase in both cardiac output and blood volume, as well as a decrease in blood pressure [7]. Additionally, adaptive changes in blood components and large vessels predispose pregnant women to conditions such as thromboembolism and arterial dissection [8]. This combination of physiological changes can destabilize underlying heart conditions and lead to life-threatening situations for the mother–fetus dyad.

Severe Maternal Morbidity (SMM) includes MM and Maternal Near Miss (MNM). The term MNM emerged in the context of reproductive health over the past two decades and, although not a new concept, it remains underrecognized [9]. Since 2009, the World Health Organization (WHO) has defined MNM as “a woman who nearly died but survived a complication that occurred during pregnancy, childbirth, or within 42 days of termination of pregnancy,” with well-established clinical, laboratory, and management criteria [10]. Maternal and fetal morbidity and mortality depend on the type and severity of the underlying heart disease. Furthermore, disparities between developed and emerging countries are expected due to differences in the epidemiology of maternal heart disease and in access to healthcare services [7,8].

In the Brazilian Network for Surveillance of SMM, heart disease was significantly associated with MM (prevalence ratio of 3.7 when comparing women with and without heart disease), with pre-existing conditions accounting for 82.6% of cases [6]. MM among women with heart disease reached 4.8% (14/293) [11], higher than the rates reported by Huisman et al. [12] in the Netherlands, where an incidence of three deaths due to heart disease per 100,000 births was identified among 97% of all deliveries in the country from 2004 to 2006.

Pregnancy represents a significant burden on the circulatory system, which explains the greater propensity of women with heart disease to experience clinical deterioration and increased rates of complications during this period. Identifying the profile of pregnant women with heart disease, maternal outcomes, and associated factors in a referral hospital may help clarify how heart disease impacts the pregnancy–puerperal period, support the development of targeted interventions during the reproductive cycle, improve quality of care, and enhance patient safety. Moreover, these findings may contribute to the creation of evidence-based guidelines and public policies aimed at reducing SMM.

Thus, the objectives of this study were to verify the SMM rate in patients with heart disease and to compare it among WHO classification groups, as well as to explore clinical and obstetric variables associated with SMM through descriptive comparisons.

## 2. Methods

This was a retrospective cohort study conducted in the Department of Obstetrics at the Paulista School of Medicine-Federal University of São Paulo (EPM-UNIFESP), which carries out its clinical activities at Hospital São Paulo (HSP). HSP is one of Brazil’s federal teaching hospitals and serves as a referral center for the care of pregnant women with heart disease [13]. This study was approved by the Ethics Committee of UNIFESP on 31 January 2019 (CAAE 04379318.9.0000.5505).

The inclusion criteria were as follows: patients with confirmed pregnancy, diagnosis of heart disease, and procedures in the obstetric center of HSP between January 2008 and December 2017. Exclusion criteria were as follows: patients with incomplete medical records, undetermined delivery outcomes, or whose diagnosis were not confirmed after delivery. Data collection was performed by the principal investigator through a review of medical records, using the Manual Data Collection Form from the National Network for Surveillance of Severe Maternal Morbidity (SMM) [14].

Although 310 women were included, some had more than one hospitalization during pregnancy, resulting in a total of 345 clinical records analyzed. Each hospitalization was considered a separate observation for the evaluation of SMM events, given the potential for new complications.

The following variables were collected: sociodemographic data, obstetric characteristics, pre-existing maternal conditions, features of heart disease, occurrence of life-threatening conditions (including hemorrhagic, hypertensive, and clinical complications), criteria for severe management (such as blood transfusion and admission to the intensive care unit), near miss events (based on clinical, laboratory, and management criteria), and maternal deaths.

Heart diseases were classified according to the modified WHO classification, ranging from Class I to Class IV, based on functional cardiac capacity and the patient’s presenting symptoms:

- Class I: Heart diseases without limitations;
- Class II: Heart diseases causing mild limitations but generally well-tolerated during pregnancy;
- Class III: Heart diseases causing significant limitations, with pregnancy posing risks;
- Class IV: Severe heart diseases where the patient cannot perform any physical activity without discomfort; in such cases, pregnancy is high-risk, requiring intensive care.

An additional category, Class II/III, was included to represent moderate to significant limitations, with symptoms such as dyspnea or fatigue during mild to moderate exertion. This classification indicates an elevated risk of complications during pregnancy and necessitates close monitoring [15].

Data were stored in Microsoft Excel 2016 spreadsheets (Microsoft Corp., Redmond, WA, USA) and analyzed using the Statistical Package for the Social Sciences, version 20.0 (SPSS Inc., Chicago, IL, USA). For the descriptive analysis of numerical variables, the mean, standard deviation, median, minimum, and maximum values were calculated. For categorical variables, absolute numbers and percentages were reported. To compare severe maternal morbidity among women with heart disease according to WHO classification, the Chi-square test or the Likelihood Ratio test was applied. A significance level of 5% (*p* < 0.05) was adopted for all statistical analyses.

Health indicators were calculated based on metrics recommended by the WHO Department of Reproductive Health and Research [16], including the following:

- Maternal Near Miss ratio (MNMR): the number of MNM cases per 1000 live births;
- Severe Maternal Morbidity ratio (SMMR): the number of MNM cases plus maternal deaths per 1000 live births;
- Maternal Near Miss/Maternal Mortality ratio (MNM/MM): the number of MNM cases per maternal death.

## 3. Results

**Figure 1:**
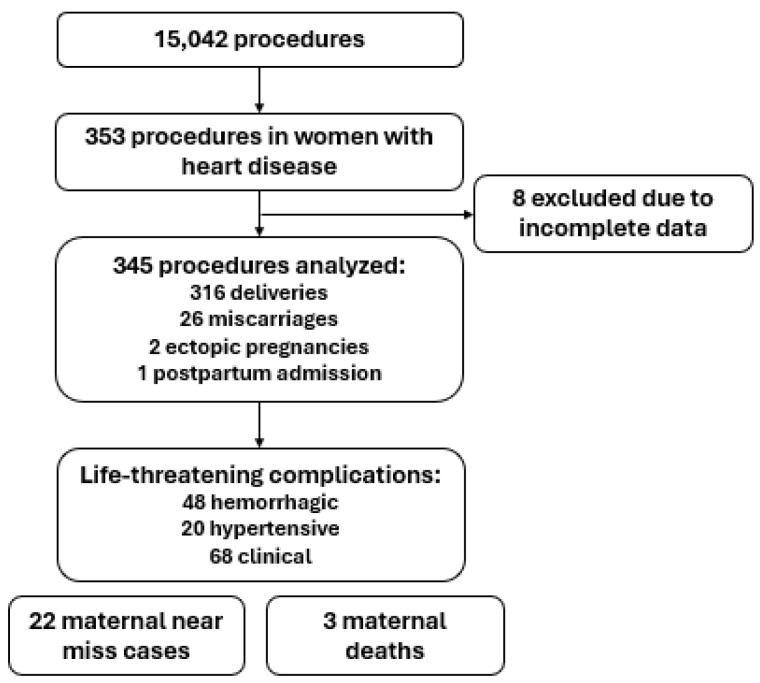
Flowchart of the included patients.

The study population comprised 345 procedures performed on 310 women with heart disease, with a mean age of 29.1 ± 7.29 years (Figure 1). Most participants self-identified as white (58.8%), had completed high school (35.9%), and were married (71.6%) (Table 1). In addition to heart disease, 9.6% of women had systemic hypertension and 9.3% had diabetes mellitus. Approximately 25% (*n* = 86) had other pre-existing conditions, most commonly pulmonary diseases (*n* = 26; 30.26%).

The mean gestational age at admission and delivery was 36.9 ± 3.22 weeks and 37.5 ± 2.66 weeks, respectively. The mean number of pregnancies per woman was 2.43 ± 1.52. Regarding the current delivery, more than half of the women underwent cesarean section (*n* = 176; 55.7%), followed by vaginal deliveries (*n* = 113; 32.8%).

According to the WHO classification of heart disease, most women were classified as Class II/III (*n* = 109; 31.6%), followed by Class I (*n* = 92; 26.7%), Class II (*n* = 60; 17.4%), Class IV (*n* = 45; 13.0%), and Class III (*n* = 39; 11.3%).

Among the 345 hospitalizations analyzed, 13.9% (*n* = 48) of patients experienced hemorrhagic complications, 19.7% (*n* = 68) clinical complications, and 5.8% (*n* = 20) hypertensive complications. The most frequent clinical complication was decompensation of the underlying heart disease (16.6%). Regarding severe management conditions (*n* = 109; 31.6%), the most common were prolonged hospitalization (25.2%) and intensive care unit (ICU) admission (11.9%).

A total of 6.4% (*n* = 22) of patients experienced MNM events. Among these, 2.9% (*n* = 10) met clinical criteria, most frequently abnormal respiratory rate (>40 or <6) and shock (1.4%), followed by cyanosis (1.2%). Laboratory criteria were observed in 4.3% (*n* = 15), and management criteria in 3.5% (*n* = 12), primarily the use of vasoactive drugs (2.3%) and intubation/ventilation for ≥60 min not related to anesthesia (2.0%).

Beyond delivery-related hospitalizations, 18.3% of patients experienced additional hospitalizations during pregnancy, primarily due to cardiac decompensation (65.1%). The median length of stay was 5 days (IQR 4.0–7.0). The SMM rate was 2.8 per 1000 live births, and the MNM-to-maternal mortality (MNM/MM) ratio was 7.3. In total, three patients (0.9%) died, resulting in a maternal mortality ratio of 34.3 per 100,000 live births during the study period.

The association between WHO maternal risk classification and outcomes—such as delivery mode, life-threatening complications, and MNM—is presented in Table 2. Women classified as WHO Class III and IV exhibited higher rates of miscarriage, clinical complications, severe management conditions, and MNM compared to those in lower-risk categories (*p* < 0.0001). Specifically, women in Class IV presented the highest proportions of clinical complications, severe management criteria, and MNM, reflecting the increased risk associated with more severe cardiac conditions.

## 4. Discussion

This study found an incidence of SMM of 2.8 per 1000 live births among pregnant women with heart disease, with significantly higher rates among those classified as WHO Classes III and IV. These findings highlight the critical need for early diagnosis and multidisciplinary care in this population. Effective management includes tailored medical therapy, continuous cardiac monitoring, and, in selected cases, surgical intervention. Patient education on warning signs and the importance of prenatal follow-up is also essential for improving maternal and neonatal outcomes [16].

Cardiac decompensation was the most frequent life-threatening complication, which is expected considering the hemodynamic stress imposed by pregnancy. Although the majority of women did not experience hemorrhagic or hypertensive complications, the implementation of preventive strategies—such as active third-stage management—may have contributed to these lower rates [11]. Nonetheless, heart failure remains the predominant challenge in this high-risk population.

One of the major difficulties in managing pregnant women with heart disease lies in the nonspecificity of early symptoms. Dyspnea on exertion, palpitations, and peripheral edema can be easily misattributed to physiological changes during pregnancy, delaying diagnosis and increasing the likelihood of maternal decompensation [15]. This reinforces the need for specialized prenatal care capable of differentiating normal from pathological findings during pregnancy.

The near miss (MNM) incidence in our study (6.4%) exceeded the national average reported by the Brazilian Network for Surveillance of SMM (MNM ratio 0.5 per 1000 live births), which likely reflects the severity and complexity of cases treated at our referral center [11]. The MNM-to-maternal mortality ratio (MNM/MM = 7.3) observed in our population suggests that timely interventions prevented death in many life-threatening situations. This ratio is widely used as a proxy for quality of care in obstetric emergencies.

Our study population was relatively young, with a mean age of 29.1 years, consistent with other reports on heart disease in pregnancy, which show that these conditions commonly occur in women of reproductive age [17,18,19]. Most women had completed high school, were married, and self-identified as white—demographic characteristics that may reflect our institutional patient profile. However, social support and education levels are recognized as factors that influence access to prenatal care and adherence to clinical recommendations [19,20,21].

A substantial proportion of women had no additional comorbidities, but hypertension and diabetes mellitus were the most common among those with pre-existing conditions. Hypertension is a recognized risk factor for preeclampsia, eclampsia, and other complications [22], while diabetes contributes to long-term cardiovascular risk through its effects on endothelial function and vascular integrity [23,24].

Clinical guidelines suggest vaginal delivery as the preferred mode in women with stable cardiac function due to lower risks of hemorrhage and infection. Nonetheless, the high cesarean rate in our cohort may reflect the need for individualized decision-making in more severe cases [24]. Most women were classified as WHO Class II/III, though a significant number were in Class III and IV, requiring close surveillance and tailored interventions [25,26,27].

The WHO classification has proven effective for stratifying maternal risk and guiding management, particularly in predicting complications such as heart failure and arrhythmias [24,28,29]. In our cohort, women classified as Class III and IV had significantly higher rates of miscarriage, clinical complications, severe management needs, and MNM. These findings are consistent with the stratification proposed in the original WHO framework.

A multicenter study involving 27 Brazilian hospitals found that 15% of women with heart disease experienced MNM [11], further confirming the elevated risk profile in this group. Rapid intervention—such as ICU care, hemodynamic support, or timely delivery—can be the difference between near miss and death. Educating patients about symptoms and ensuring timely access to specialized care are fundamental [12].

International comparisons demonstrate lower maternal mortality rates in high-income settings [12,15], where health systems offer greater access to preconception counseling, contraception, and multidisciplinary care. Structural inequalities in Brazil, including gaps in healthcare access and resource allocation, may contribute to the relatively higher rates of adverse outcomes [30,31].

Historical data from our institution (1979–1989) reported a maternal mortality rate of 1.7% among women with heart disease [31], while our current rate of 0.9% reflects improvement over time. Nevertheless, our findings still suggest higher morbidity compared to international reports. In particular, women in WHO Class III and IV should receive thorough counseling regarding the risks of pregnancy, including the possibility of termination where legally permitted. If pregnancy continues, these women require close follow-up, often involving prolonged hospitalization, medication adjustments, and planning for early delivery and neonatal care [8,32,33,34].

The most recent studies have shown that women in WHO Classes I and II experience outcomes comparable to the general obstetric population, whereas those in Classes II/III to IV face a significantly elevated risk. For instance, women in WHO II/III–IV have an adjusted RR of 5.67 for SMM and 18.07 for maternal death compared to women without heart disease [35,36,37].

These findings align closely with the results of our study, in which women classified as WHO Classes III and IV had the highest rates of miscarriage, clinical complications, severe management conditions, and MNM. Our data reinforce the predictive value of the WHO classification and support its use in clinical practice, as endorsed by the American Heart Association and the Society for Maternal-Fetal Medicine [36]. The correlation between a higher WHO class and more adverse outcomes—including increased cesarean rates and maternal mortality—further emphasizes the importance of individualized counseling, intensive surveillance, and preconception risk assessment in this high-risk population [35,37,38].

Finally, although our study has limitations—such as its single-center design, retrospective methodology, and data collection challenges during the COVID-19 pandemic—it has several strengths. It evaluates a large, high-risk cohort over an extended period and applies standardized criteria to explore the relationship between heart disease severity and maternal outcomes.

## 5. Conclusions

This study demonstrated that the SMM ratio among pregnant women with heart disease was 2.8 cases per 1000 live births and was significantly associated with higher maternal risk, particularly in WHO Classes III and IV.

## Figures and Tables

**Table 1:** Sociodemographic and clinical characteristics of the study population.

Variable	Total *n* (%)
Age	
Mean (Standard Deviation)	29.11 (7.29)
Median (Minimum–Maximum)	29 (13–45)
Ethnic group	
Black	30 (8.7)
White	203 (58.8)
Asian	1 (0.3)
Mixed ethnicity	111 (32.2)
Education	
Illiterate	4 (1.1)
Incomplete primary education	74 (21.5)
Primary education	28 (8.1)
Incomplete high school	47 (13.6)
High school	124 (36)
Incomplete college	27 (7.8)
College	23 (6.7)
Marital Status	
Married/Cohabitating	247 (71.6)
Single	94 (27.2)
Separated/Divorced	4 (1.2)
Pre-existing maternal conditions	
Chronic hypertension	33 (9.6)
Obesity	18 (5.2)
Diabetes mellitus	32 (9.3)
Smoking	25 (7.2)
Kidney disease	6 (1.7)
Anemia	8 (2.3)
Others	86 (24.9)

**Table 2:** Pregnancy outcomes and maternal morbidity according to WHO maternal risk classification.

	WHO Maternal Risk Classification					Total
	I (*n* = 83)	II (*n* = 59)	II/III (*n* = 104)	III (*n* = 29)	IV (*n* = 41)	(*n* = 316)
Mode of Delivery						
Vaginal delivery	30 (36.1)	27 (45.8)	51 (49.0)	14 (48.3)	18 (43.9)	140 (44.3)
Cesarean section	53 (63.9)	32 (54.2)	53 (51.0)	15 (51.7)	23 (56.1)	176 (55.7)
Hemorrhagic Complications	9 (10.8)	5 (8.5)	15 (14.4)	9 (31.0)	10 (24.4)	48 (15.2)
Hypertensive Complications	5 (6.0)	4 (6.8)	8 (7.7)	0 (0.0)	3 (7.3)	20 (6.3)
Clinical Complications	2 (2.4)	3 (5.1)	22 (21.2)	12 (41.4)	29 (70.7)	68 (21.5)
Potentially Life-Threatening Conditions	11 (13.3)	10 (16.9)	29 (27.9)	26 (89.7)	33 (80.5)	109 (34.5)
Near Miss	1 (1.2)	2 (3.4)	6 (5.8)	2 (6.9)	11 (26.8)	22 (7.0)
Clinical Criteria	0 (0.0)	1 (1.7)	1 (1.0)	0 (0.0)	8 (19.5)	10 (3.2)
Laboratory Criteria	1 (1.2)	1 (1.7)	5 (4.8)	1 (3.4)	7 (17.1)	15 (4.7)
Management Criteria	0 (0.0)	2 (3.4)	1 (1.0)	1 (3.4)	8 (19.5)	12 (3.8)

## Data Availability

Data available on request from the authors.
